# Paediatric aortic valve replacement using decellularized allografts: a multicentre update following 143 implantations and five-year mean follow-up

**DOI:** 10.1093/ejcts/ezae112

**Published:** 2024-03-26

**Authors:** Alexander Horke, Dmitry Bobylev, Murat Avsar, Tomislav Cvitkovic, Bart Meyns, Filip Rega, Mark Hazekamp, Robert Cesnjevar, Martin Schmiady, Brigitte Staebler, Oliver Dewald, Anatol Ciubotaru, Ina Michel-Behnke, Daniel Zimpfer, Ramadan Jashari, Dietmar Boethig, Serghei Cebotari, Philipp Beerbaum, Igor Tudorache, Samir Sarikouch

**Affiliations:** Department for Cardiothoracic, Transplant, and Vascular Surgery, Hannover Medical School, Hannover, Germany; Department for Cardiothoracic, Transplant, and Vascular Surgery, Hannover Medical School, Hannover, Germany; Department for Cardiothoracic, Transplant, and Vascular Surgery, Hannover Medical School, Hannover, Germany; Department for Cardiothoracic, Transplant, and Vascular Surgery, Hannover Medical School, Hannover, Germany; Department of Cardiac Surgery, Katholieke Universiteit Leuven, Belgium; Department of Cardiac Surgery, Katholieke Universiteit Leuven, Belgium; Department of Cardiothoracic Surgery, Leiden University Medical Center, Netherlands; Division of Congenital Cardiovascular Surgery, University Children’s Hospital, Zurich, Switzerland; Division of Congenital Cardiovascular Surgery, University Children’s Hospital, Zurich, Switzerland; Department of Cardiac Surgery, Sana Herzchirurgie, Stuttgart, Germany; Division of Pediatric Cardiac Surgery, University of Erlangen, Erlangen, Germany; Cardiac Surgery Center, State Medical and Pharmaceutical University, Chisinau, Moldova; Department of Pediatric Cardiology, Medical University of Vienna, Austria; Department of Cardiac Surgery, Medical University of Vienna, Austria; European Homograft Bank, Clinique Saint-Jean, Brussels, Belgium; Department for Cardiothoracic, Transplant, and Vascular Surgery, Hannover Medical School, Hannover, Germany; Department for Pediatric Cardiology and Intensive Care, Hannover Medical School, Germany; Department of Cardiac Surgery, Institute for Cardiac Surgery and Interventional Cardiology, Luxembourg, Luxembourg; Department for Pediatric Cardiology and Intensive Care, Hannover Medical School, Germany; Department for Cardiothoracic, Transplant, and Vascular Surgery, Hannover Medical School, Hannover, Germany; Clinic for Cardiac Surgery, University Heart Center Zurich, Zurich, Switzerland; Department for Cardiothoracic, Transplant, and Vascular Surgery, Hannover Medical School, Hannover, Germany

**Keywords:** Children, Aortic valve disease, decellularization, Allografts

## Abstract

**OBJECTIVES:**

Decellularized aortic homografts (DAH) were introduced in 2008 as a further option for paediatric aortic valve replacement (AVR).

**METHODS:**

Prospective, multicentre follow-up of all paediatric patients receiving DAH for AVR in 8 European centres.

**RESULTS:**

A total of 143 DAH were implanted between February 2008 and February 2023 in 137 children (106 male, 74%) with a median age of 10.8 years (interquartile range 6.6–14.6). Eighty-four (59%) had undergone previous cardiac operations and 24 (17%) had undergone previous AVR. The median implanted DAH diameter was 21 mm (interquartile range 19–23). The median operation duration was 348 min (227–439) with a median cardiopulmonary bypass time of 212 min (171–257) and a median cross-clamp time of 135 min (113–164). After a median follow-up of 5.3 years (3.3–7.2, max. 15.2 years), the primary efficacy end-points peak gradient (median 14 mmHg, 9–28) and regurgitation (median 0.5, interquartile range 0–1, grade 0–3) showed good results but an increase over time. Freedom from death/explantation/endocarditis/bleeding/thromboembolism at 5 years were 97.8 ± 1.2/88.7 ± 3.3/99.1 ± 0.9/100 and 99.2 ± 0.8%, respectively. Freedom from death/explantation/endocarditis/bleeding/thromboembolism at 10 years were 96.3 ± 1.9/67.1 ± 8.0/93.6 ± 3.9/98.6 ± 1.4 and 86.9 ± 11.6%, respectively. In total, 21 DAH were explanted. Seven were replaced by a mechanical AVR, 1 Ross operation was performed and a re-do DAH was implanted in 13 patients with no redo mortality. The calculated expected adverse events were lower for DAH compared to cryopreserved homograft patients (mean age 8.4 years), and in the same range as for Ross patients (9.2 years) and mechanical AVR (13.0 years).

**CONCLUSIONS:**

This large-scale prospective analysis demonstrates excellent mid-term survival using DAH with adverse event rates comparable to paediatric Ross procedures.

**Figure:**
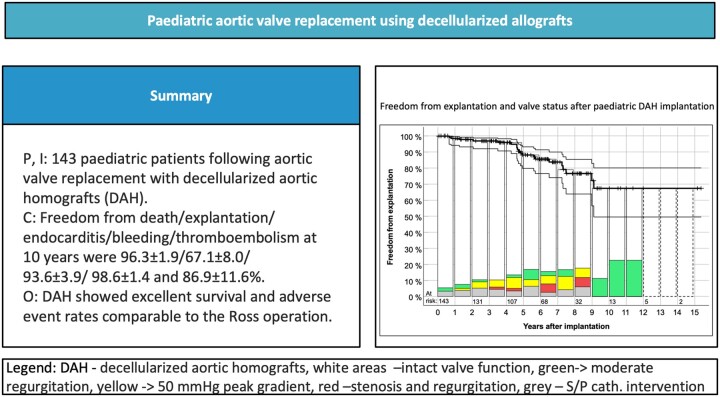

## INTRODUCTION

Paediatric aortic valve replacement (AVR) procedures can essentially be divided into 2 categories. One category comprises several options for aortic root replacement, which come at the cost of a longer and more complex operation, and the majority of which include coronary reimplantation. The 2nd category includes all intra-annular valve replacement options, which require sufficient subvalvular and valvular dimensions. Mechanical prostheses constitute the only viable intra-annular aortic valve for children as all xenogeneic biological prostheses bear the inherent risk of rapid degeneration and subsequent myocardial failure [1].

Paediatric Ross operations, predominantly performed using the full-root technique in childhood, have demonstrated excellent results in specialized institutions [2–4]. In young children, it nevertheless has a considerable operative mortality ranging up to 24% for neonates, which is, of course, biased by the severe valvular aortic disease present in these patients [5]. Conducting a Ross procedure in very young patients also aggravates the rate for redo procedures on the right ventricular outflow tract, which will inevitably result in numerous replacement procedures over a lifetime [6, 7]. Aortic root stabilizing techniques in younger children can decrease the growth potential of the pulmonary autograft and are therefore infrequently used. An associated aortic root dilatation is one potential long-term complication, which also leads to more reoperations [8, 9]. Ross procedures in children during their 2nd decade have a very low mortality of ∼1% [5]. Autograft dilatation in older children is also less frequent due to modern root stabilizing techniques and larger pulmonary valve substitutes reduce the need for reoperation on the 2nd semilunar valve affected by the Ross procedure [10].

Homografts are the main 2nd option for full aortic root replacement as xenogeneic full aortic root replacement has shown poor results in children, which is thought to be due to the high immunogenicity of the porcine grafts [11]. Homografts provide the same excellent haemodynamic physiology as pulmonary autografts, but do also elicit a considerable immune response [12]. Decellularized aortic homografts (DAH) have the potential to be less immunogenic [13] and early results have demonstrated significantly better freedom from adverse events than conventional cryopreserved aortic homografts. DAH results are comparable to those for Ross operations, achieving normal haemodynamics without the need to remove the pulmonary valve [14, 15].

Decellularized homografts may therefore offer an alternative for those patients, who are not good Ross candidates due to multiple previous aortic root procedures or an incompetent pulmonary valve.

The aim of this study is to present an update on the current results of paediatric DAH implantation and to compare these results with contemporary data on the Ross procedure and other AVR options for children.

## MATERIALS AND METHODS

### Study setting

The study design was a prospective, multicentre follow-up of all paediatric patients receiving DAH for AVR in 8 European centres (Hannover, Leuven, Leiden, Zürich, Chisinau, Stuttgart, Erlangen and Vienna); 40/137 patients analysed participated in the prospective ARISE trial (ClinicalTrials.gov, NCT02527629). Patients were not included consecutively and patient selection was based on decision of the respective centre, the availability of an appropriate homograft and patient consent.

The primary end-points were periprocedural complications (all-cause mortality, major stroke, life-threatening or disabling bleeding, acute kidney injury requiring renal replacement therapy, myocardial infarction, major vascular complications), heart valve dysfunction and repeat procedure for valve-related dysfunction (surgical or interventional therapy). An indication for AVR according to the current clinical guidelines of the European Association for Paediatric Cardiology was the key inclusion criterion. Children with active endocarditis were not included as recommended by the DAH instructions for use. Approval for this non-interventional follow-up study was granted by Hannover Medical School Ethics Committee (No. 1503-2012) at the study outset, and informed consent was obtained appropriately from all parents at the respective institutions.

Surgical procedures were performed according to locally established standard procedures under cardiopulmonary bypass. Postoperatively, patients were recommended aspirin/acetylsalicylic acid at 2–3 mg per kilogram per day for 3–6 months and in some adolescent patients, warfarin therapy was recommended for 2 months followed by continued acetylsalicylic acid medication. All DAH implantations were performed as a root replacement with coronary reimplantation, and without any significant reinforcement procedures. Patients were followed annually by echocardiography at their respective centres or by their resident paediatric cardiologist. There was a 100% follow-up of patients for events as freedom from death, endocarditis and reoperation. We used the latest echo data, which was available at the time of the analysis in February 2023 (72.7% completeness for 2022 and 2023 data).

### Homograft procurement and processing

Homografts were procured in line with the current European Directive 2004/2023, as amended, via 3 different tissue banks (European Homograft Bank, Brussels, Dr Ramadan Jashari; German Society for Tissue Transplantation—DGFG, Hannover, M. Börgel; EuroTissue Bank, Rotterdam, A. van den Bogaerdt) and shipped to Hannover for processing at Corlife oHG (www.corlife.eu). DAH was authorized by the German competent authority as ‘Cell-free aortic heart valve, Arise AV’, # PEI.G.11766.0.1. The processing of each homograft comprises ∼30 different steps using a detergent-based, non-cryopreservation approach as described previously [16]. Microbiological assessment was performed as part of the incoming inspection, and both during and after processing with a final 14-day quarantine. Each homograft was assessed histologically following processing, and the residual dsDNA content was measured before and after processing prior to final release. Reference samples of all homografts were stored for at least 1 year in accordance with the German law.

### Statistics

Summaries of the numeric data are given as means and standard deviation or median and interquartile range, as appropriate. The normal distribution of factors was assessed using Kolmogorov–Smirnov. Categorical variables are given as counts and percentages. The proportion of explanted and dysfunctional grafts over time was calculated and a peak echocardiographic gradient of ≥50 mmHg and regurgitation greater than or equal to moderate were defined as dysfunctional. Time-related events, such as freedom from explantation and degeneration, were evaluated according to Kaplan–Meier. We did not assess the competing risks of death and heart valve dysfunction as mortality was very low.

To describe the course of valve performance over time, we used 1 valve status observation per postoperative year, classifying the valve as intact, insufficient, stenotic, insufficient and stenotic or status postintervention. Frequency of each functional status was calculated for each year. Since the status frequencies applied only for non-explanted conduits, these frequencies were multiplied by the fraction of conduits that were not explanted by the middle of the relevant year. Hence, the functional valve status frequencies presented here refer to all initially implanted DAH in the respective period.

We calculated perioperative mortality and the annually reported adverse rates for events such as late death, reoperation or reintervention, valve degeneration, thrombotic and bleeding events and endocarditis for all paediatric DAH implanted to date. DAH data were compared with the results of a recent large-scale meta-analysis for paediatric AVR (median follow-up 5.9 years), which included studies on 3468 paediatric Ross patients, 799 children with mechanical AVR and 517 patients undergoing AVR using a standard cryopreserved homograft [17].

Binomial 95% confidence limits were calculated (with MedCalc^®^, MedCalc^®^ Statistical Software version 22.016, MedCalc Software Ltd, Ostend, Belgium; https://www.medcalc.org; 2023) for the adverse event rates shown in Table 2, assuming a decrease of initial patient numbers proportional to the decrease observed for the DAH population. Non-overlapping confidence limits indicate statistically significant differences according to a *P*-value <0.05%.

**Table 1:** Patient characteristics for the paediatric DAH cohort and the total ARISE Registry cohort (all DAH)

	Paediatric AVR, *n* = 143	All DAH *n* = 358
Implantation period	2008–2023	2008–2023
Age at implantation (years)	10.8 [6.6–14.6]	23.9 [12.5–44.8]
Follow-up (years)	5.3 [3.3–7.2]	5.0 [3.1–6.5]
Total follow-up (years)	756	1748
Sex (male)	106 (74%)	250 (70 %)
Number of previous operations		
0	59	200
1	47	90
2	24	46
>2	13	22
Type of previous procedures		
1 × aortic valve replacement	19	49
2 × aortic valve replacement	4	11
3 × aortic valve replacement	1	2
Catheter-based intervention	68	92
Aortic valve repair	28	40
Allograft diameter (mm), median [IQR]	21 [19–23]	23 [21–25]
10–18	26	28
19–22	76	127
23–29	41	203
Implantation time (min)		
Total operation	348 [227–439]	303 [244–400]
Cardiopulmonary bypass	212 [171–257]	168 [132–220]
Cross clamp	135 [113–164]	120 [97–146]
Latest echocardiography		
Aortic annulus (mm)	20.5 (3.7)	22.1 (3.8)
Aortic annulus, z-score	0.02(1.50)	0.18 (1.47)
Effective orifice area (cm^2^)	2.36 (0.84)	2.95 (0.83)
Peak gradient (mmHg)	14 [9–28]	11.6 [7.8–19.4]
Regurgitation (grade 0–3)	0.5 [0–1]	0.5 [0–1]
LV ejection fraction (%)	64.7 [58.5–68.5]	64.0 [59.5–68.0]

Mean and standard deviation are shown in round brackets for normally distributed factors, median and IQR are shown in square brackets for factors with no normal distribution.

AVR: aortic valve replacement; DAH: decellularized aortic homografts; IQR: interquartile range; LV: left ventricular.

**Table 2:** Observed early and late adverse events for DAH in children with comparison to reported results of paediatric Ross procedures, mechanical AVR and standard allograft implantation in children [17]

	DAH (*n* = 143)	Ross (*n* = 3468)	Mechanical (*n* = 799)	Allograft (*n* = 517)
Early death (%)	0.70	3.72	6.95	10.55
Early reintervention (%)	4.90	4.69	4.20	2.83
Early stroke (%)	0.0	0.76	1.45	0.52
Early pacemaker (%)	2.10	3.17	3.37	2.13
**Sum of early adverse events (%/year)**	**7.61**	**12.34**	**15.97**	**16.02**
Late mortality (%/year)	0.40	0.51	0.99	1.38
Any reoperation/intervention (%/year)	3.41	3.42	1.18	4.78
Endocarditis (%/year)	0.27	0.37	0.34	0.34
Thromboembolism	0	0.13	0.42	0.09
Valve thrombosis (%/year)	0	0.19	0.33	0.11
Bleeding (%/year)	0	0.09	0.32	0.09
Stroke/TIA (%/year)	0.14	0.09	0.43	0.11
**Sum of late events**	**4.22**	**4.80**	**4.01**	**7.01**

Ross results include right ventricular procedures.

AVR: aortic valve replacement; DAH: decellularized aortic homograft; TIA: Transient ischemic attack.

SPSS 28 (IBM Corporation, Somer, NY), R (R version 4.2.2; 2022–10-31 ucrt, The R Foundation for Statistical Computing) and RStudio (RStudio Team 2020, PBC, Boston, MA) and EZR Version 1.61 were used for the analyses and the graphical result illustrations.

## RESULTS

### Perioperative and late outcome of paediatric decellularized aortic homografts implantation

One-hundred and forty-three DAH were implanted between February 2008 and February 2023 in 137 children (106 male, 74%) with a median age of 10.8 years [interquartile range (IQR) 6.6–14.6, mean 10.4 (standard deviation, SD 4.8 years)]. Eighty-four (59%) had undergone previous cardiac operations (47 with 1, 24 with 2, 13 with ≥3 previous operations). Twenty-four (17%) had undergone previous AVR (19 with 1, 4 with 2 and 1 with 3 previous operations).

The median implanted DAH diameter was 21 mm (IQR 19–23). The median operation duration was 348 min (IQR 227–439) with a median cardiopulmonary bypass time of 212 min (IQR 171–257) and a median cross-clamp time of 135 min (IQR 113–164). Table 1 provides the study cohort characteristics.

There was 1 death in the perioperative period: a 12-year-old girl died while on ECMO after undergoing her 4th aortic valve operation. A 2-year-old girl died due to sepsis 2 months postoperatively without any evidence of endocarditis. In addition, a heart transplantation was performed in a 2.5-year-old girl 8 months after DAH implantation due to pre-existing myocardial failure, which did not improve despite the DAH implantation and normal homograft function.

Three late deaths occurred. A 27-year-old male died in Moldavia due to a COVID19 infection 12 years after implantation with no apparent links to the function of the aortic valve. A 23-year-old male died 6 years after implantation as a result of endocarditis, which led to severe, rapidly progressing aortic regurgitation. A 2-year-old boy died of myocardial failure 1 year postoperatively following his 6th cardiac procedure for complex left heart disease. In 3/143 DAH implantations (2.1%), pacemaker implantation was subsequently necessary.

### Coronary reimplantation complications

In 8/143 paediatric DAH implantations (5.6%), with a mean patient age of 10.3 years (SD 5.3, median 12.0 years, IQR 5.6–14.3), significant problems occurred during coronary reimplantation in 7 patients, including 1 DAH redo procedure; 4/7 patients had 2 or more previous cardiac surgeries.

Patients 1–4 received isolated proximal RCA stents to remedy persisting postoperative ECG alteration. Patient 5 received a single venous coronary artery bypass following a preparation injury to the right coronary artery.

Patient 6 received an A. mammaria-left anterior descending coronary artery bypass in addition to a RCA stent. The 12-year-old girl had undergone her 4th aortic valve operation and died from intracerebral haemorrhage while on ECMO support.

Patient 7 received an A. mammaria graft as a free graft to the left circumflex artery (LCx) due to persisting inferior LV hypo-motility at the end of the operation, which resolved after bypass grafting. A coronary angiography performed 6 months later showed a normal left ventricular function and normal LCA and LCx. The arterial bypass to the LCx was almost totally occluded, rendering a temporary LCx perfusion problem the most likely explanation for the impairment. Interestingly, the same patient required 2 stents (LCA and RCA) 5.7 years later during reoperation for DAH exchange; these postoperative episodes were most likely due to recurrent vasospasm.

Among these 7 patients, 1 death occurred (patient 6). Patient 7, who experienced coronary complications during both the initial and redo DAH implantations, suffered permanent LV function impairment (left ventricular ejection fraction 42%) due to a sub-endocardial myocardial infarction following the second operation.

### Midterm outcome of paediatric decellularized aortic homografts

Median follow-up was 5.3 years (IQR 3.3–7.2, max. 15.2 years). The primary efficacy end-points peak gradient of median 14 mmHg (IQR 9–28) and regurgitation of median 0.5 (IQR 0–1, grade 0–3) in the 5th year showed good results but an increased progression over time.

The median left ventricular ejection fraction was 64.7 (58.5–68.5) and the mean aortic valve diameter 20.5 mm (SD 3.7), with a mean z-score of 0.02 (SD 1.50) and a mean effective orifice area of 2.36 cm^2^ (SD 0.84).

Freedom from death/explantation/endocarditis/bleeding/thromboembolism at 5 years (*n* = 68) were 97.8 (SD 1.2)/88.7 (SD 3.3)/99.1 (SD 0.9)/100/99.2 (SD 0.8)%, respectively.

Freedom from death/explantation/endocarditis/bleeding/thromboembolism at 7.5 years (*n* = 32) were 97.8 (SD 1.2)/76.6 (SD 5.4)/99.1 (SD 0.9)/98.6 (SD1.4)/97.6 (SD 2.4)%, respectively.

Freedom from death/explantation/endocarditis/bleeding/thromboembolism at 10 years (*n* = 13) were 96.3 (SD 1.9)/67.1 (SD 8.0)/93.6 (SD 3.9)/98.6 (SD 1.4)/86.9 (SD 11.6)%, respectively.

In total, 21 DAH were explanted. Seven were replaced by a mechanical AVR, 1 Ross operation was performed and a redo DAH was implanted in 13 patients. The mortality was 0% in these 21 operations.

**Figure 1:**
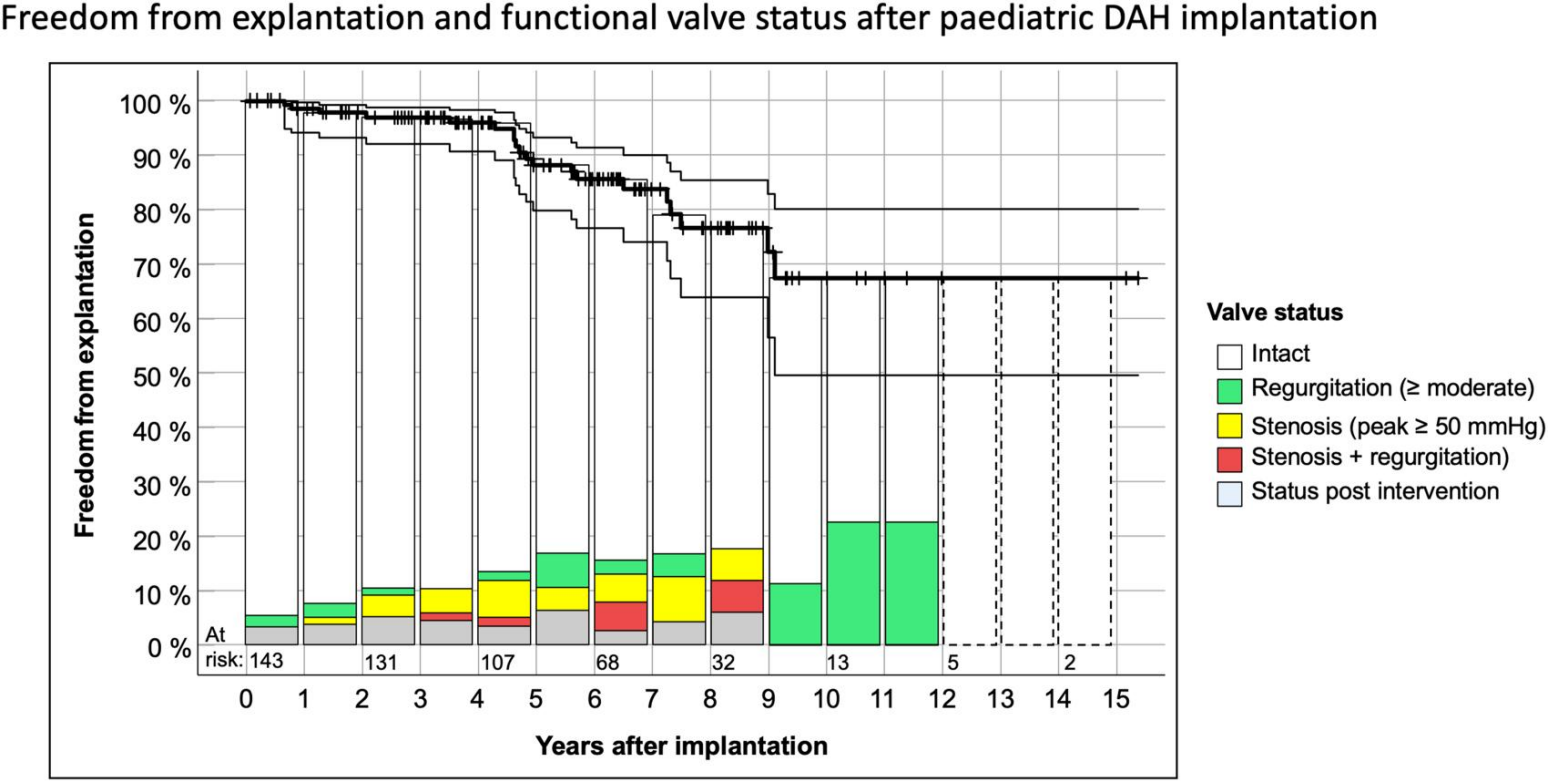
Freedom from explantation according to Kaplan–Meier with 95% confidence intervals and functional status of all implanted DAH in children. Displayed functional status frequencies refer to all DAH implanted in the respective period. Dashed boxes indicate years in which follow-ups in Moldovia could be performed only via telephone due to the COVID19 pandemic and the war in Ukraine. DAH: decellularized aortic homograft.

Figure 1 shows freedom from explantation according to Kaplan–Meier with 95% confidence intervals and the functional status of all implanted DAH in children with up to 15 years of follow-up. The dashed boxes indicate years in which follow-ups in Moldovia could be performed only via telephone due to the COVID19 pandemic and the war in Ukraine. Table 1 also provides detailed paediatric DAH data as well as data on all adults with DAH for comparison.

### Decellularized aortic homografts performance over time

**Figure 2:**
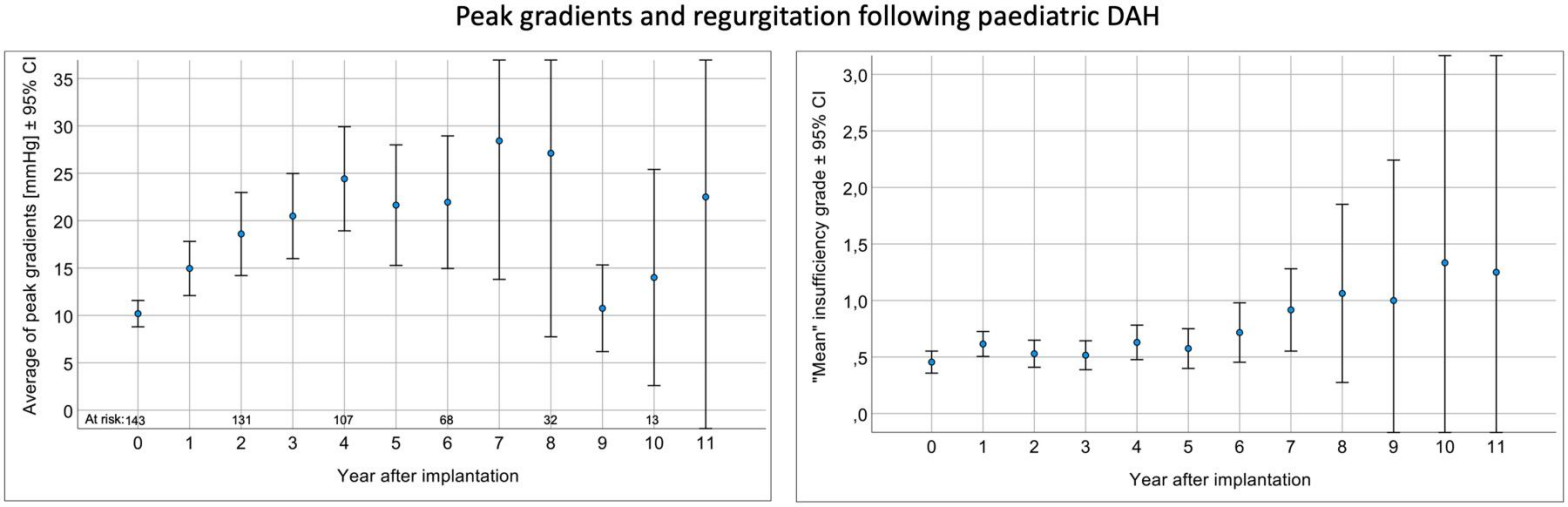
Development of aortic valve gradients and regurgitation over time following DAH. We entered 1 value per patient-follow-up year, if necessary, linearly interpolated. DAH: decellularized aortic homograft.

**Figure 3:**
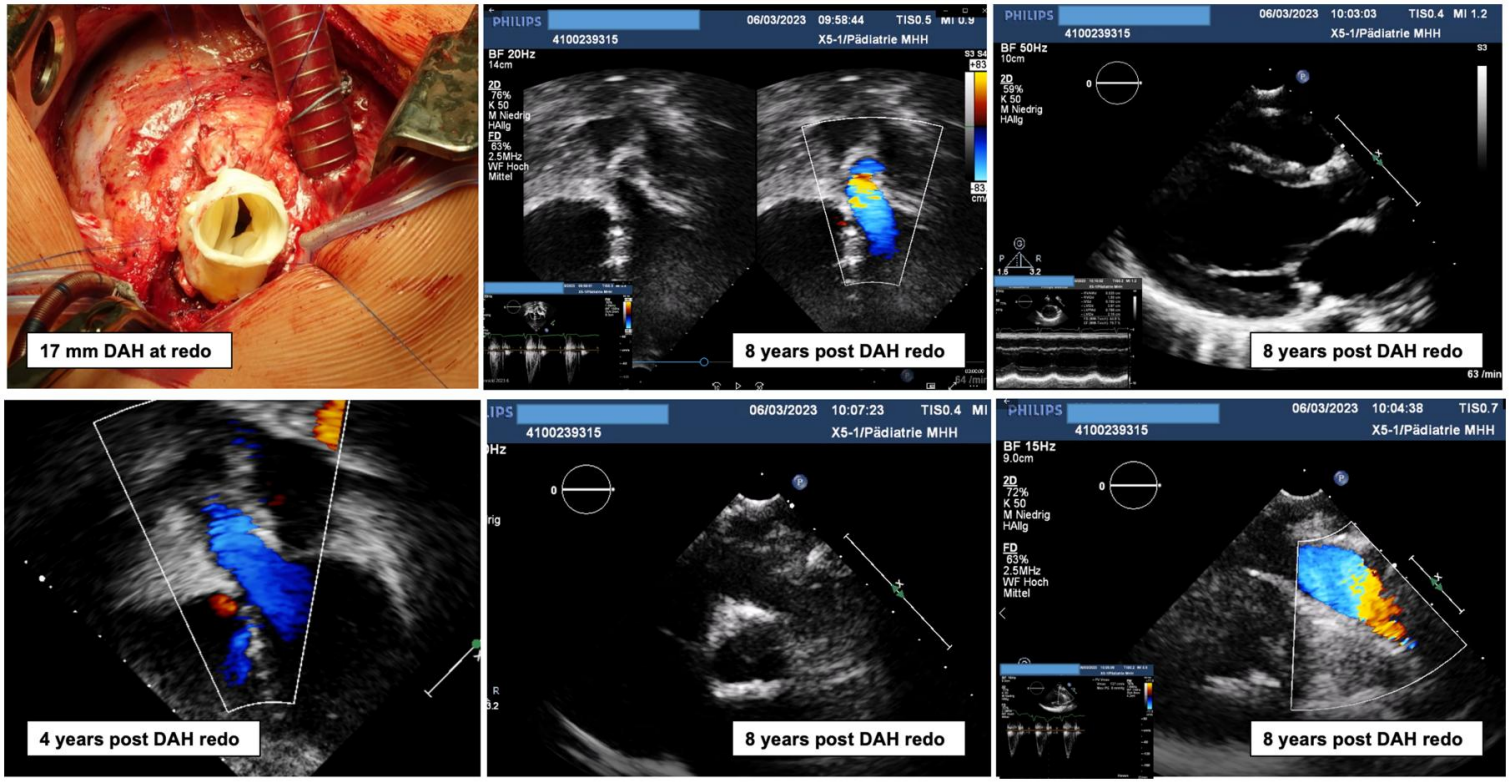
A 0.2-year-old boy, *S/P* 2 × aortic valve balloon valvuloplasty, AVR with DAH 10 mm in 2010 and redo AVR in 2015 with 17 mm DAH. Recent echo at 8 years following redo DAH implantation shows normal ventricular dimensions and function. Competent and pliable DAH cusps and a normal, untouched pulmonary valve. AVR: aortic valve replacement; DAH: decellularized aortic homograft.

Figure 2 displays the development of the peak aortic valve gradient (in mmHg) and aortic regurgitation (0: no regurgitation, 0.5: trace, 1.0: mild, 1.5: mild to moderate, 2.0: moderate, 2.5: moderate to severe and 3.0: severe) during follow-up. There was a slow increase of both haemodynamic parameters over time. Figure 3 exhibits the patient with the longest follow-up after redo DAH implantation.

Following 2 balloon dilatations, he underwent AVR with a 10 mm DAH at the age of 2 months in 2010 and a subsequent redo AVR in 2015 with a 17 mm DAH due to subvalvular stenosis leading to aortic regurgitation by Jet-lesion destruction of one cusp. The patient had normal left ventricular and homograft function 8 years after the redo procedure.

### Updated expected adverse events for contemporary aortic valve replacement options in children

**Figure 4:**
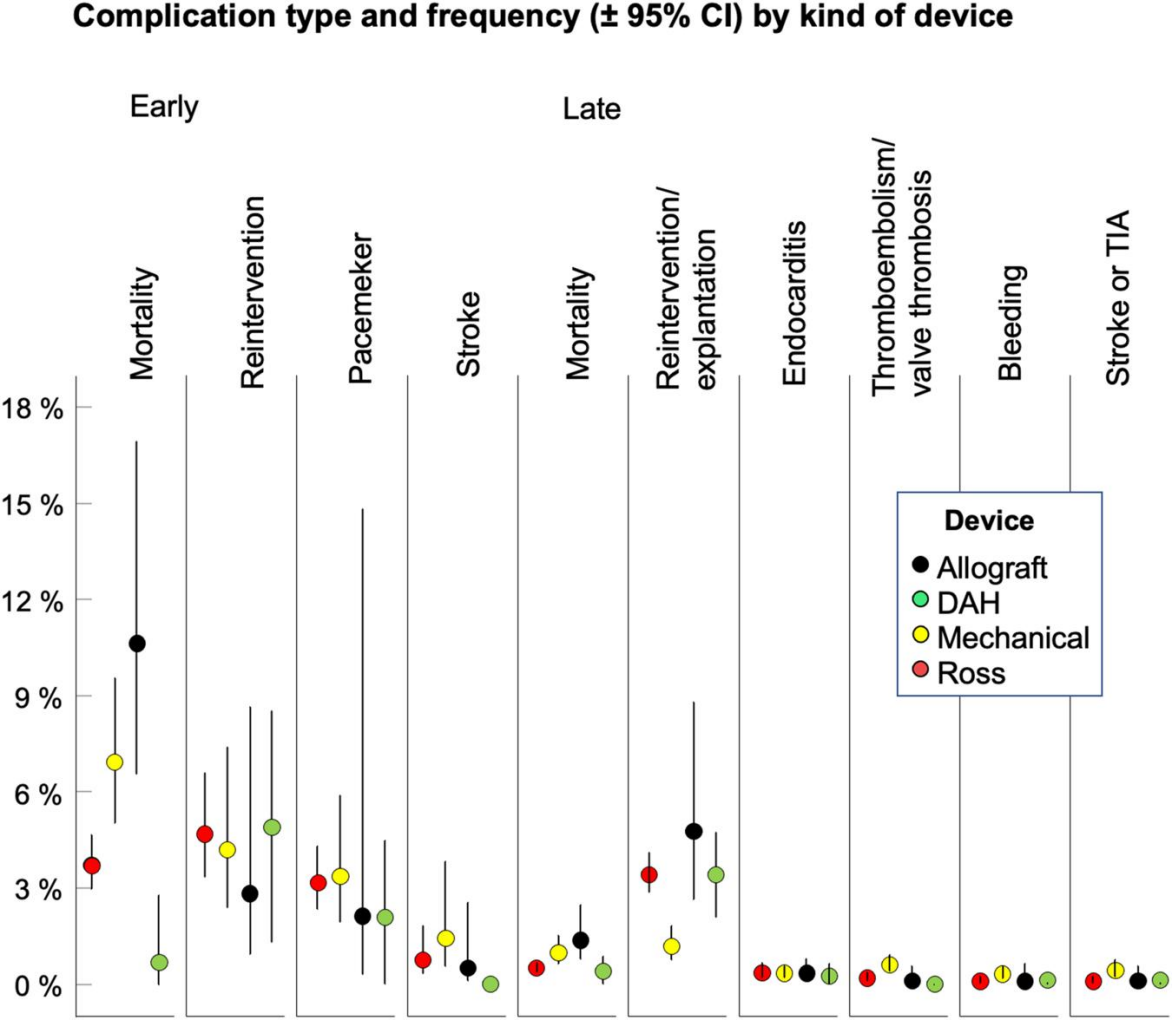
Paediatric DAH implanted to date in comparison with recently published meta-analysis data for several AVR options in children. Observed averaged rates for early and late adverse event category (± 95% confidence intervals) within the 4 different aortic valve substitutes. Ross results include right ventricular procedures [17]. DAH: decellularized aortic homograft. Data taken from: Notenboom *et al*. Eur Heart J. 2023 Jun 27.

Figure 4 and Table 2 show DAH performance in comparison with recently published meta-analysis data for several AVR options in children [17]. Ross results include right ventricular procedures.

DAH in children (mean age 10.4 years, median 10.8) demonstrated lower early mortality than mechanical AVR (mean age 13.0 years), the Ross procedure (mean age 9.2 years) and conventionally cryopreserved homografts (mean age 8.4 years). Late annual adverse events were comparable to Ross and mechanical AVR and lower than for cryopreserved homografts.

## DISCUSSION

The current study adds important information on the mid-term outcomes following AVR with DAH in children.

Freedom from death was 97.8 and 96.3% at 5 and 10 years, an outstanding result, which underlines the expertise of the participating centres. Adverse events such as reoperation, valve degeneration, endocarditis or thromboembolism after a mean follow-up of 5.3 years were comparable to the results from current paediatric Ross cohorts, despite DAH patients undergoing twice as many previous cardiac operations (59% vs 32.3%) and significantly more previous AVR (17% vs 2.3%) [10]. Intraoperative complications during coronary artery transfer occurred in 5.6% of the DAH patients, which is also comparable to the rates of 6.5% in children and 5% in young adults for coronary artery bypass grafting associated with the Ross procedure [6, 10]. Hypoplasia of the right coronary artery, an anomalous origin and mismatch between original aortic root dimensions and previous aortic root procedures were identified as risk factors for coronary complications. Overall, operative mortality was extremely low at 0.7%. There also was no mortality in reoperations following DAH, which indicates less complex reoperations compared to the sometimes challenging redo’s following conventional homografts.

The results, however, also demonstrate rising gradients and regurgitation in DAH over time leading to surgical reintervention. Despite thorough decellularization and detailed quality assurance through individual DAH testing before release, DAH appear to elicit a low-grade immune response, which, in contrast to classic T-cell mediated immune reactions, is thought to be more antibody-mediated [18]. We have shown the highly individual antibody binding towards specific decellularized homografts and are currently performing longitudinal analyses on humoral immune responses in patients following DAH implantation [13, 19]. To date, no information is available on the potential antigen(s) causing these immunologic response. Current hypotheses favour so-called matrikines, specific peptides of the extracellular matrix that may emerge after the decellularization process as a target for the host immune system [20]. Silencing strategies or intensified decellularization might provide a future option once the target antigens are known. Another hypothesis, supported by new cytokine and chemokine analyses of explanted decellularized heart valves (in preparation), points towards irregular repair mechanisms following recellularization.

In light of these results, can DAH continue to be considered as an alternative option to the Ross operation for paediatric patients? Clearly, the aim of substantial integration and recellularization of the decellularized matrix with recipient cells has not yet been sufficiently met with the current technique. Implantation of a DAH in a child will lead to later reoperation and parents must be informed accordingly. Nevertheless, DAH still can offer a valid option for patients who are not good Ross candidates, as our results demonstrate similar outcomes to the Ross operation with low mortality. DAH also provides an option in patients with a poor left ventricular function, who would then only face the risk of single semilunar valve operation. DAH may also be used to bridge patients for a certain period of time, as Ross results for older children are substantially better than those in very young patients. A pulmonary autograft transfer later in life could allow root stabilizing procedures and thereby reduce the rate of autograft dilatation in paediatric Ross patients.

Can an aortic root procedure, including an associated coronary artery transfer, be justified based on the current DAH results in children where aortic dimensions would allow the implantation of a mechanical valve? This is an important question and highlights the need to weigh up the burden of a more extensive operation, which can provide near-normal physiology without the need for permanent anti-coagulation on the one hand, and the reduced effective orifice area and risks associated with blood thinners on the other.

In our comparison of the expected adverse events for the different AVR options in childhood, mechanical valves had less events than Ross procedures, which may have been due to the higher mean age of mechanical AVR patients. There, however, is sobering data on the long-term results of mechanical AVR in childhood. An analysis of 121 patients, who received a mechanical AVR at the Boston Children′s Hospital, recorded the survival rate at 10 years as only 81.5%, and freedom from aortic valve reoperation at 10 years was 78.4% in a cohort of a median age of 16 years [21]. In a 22-year single-centre retrospective analysis by Brown *et al.*, the 10-year freedom from death was 84% in patients, who initially were evaluated as Ross candidates, but for several reasons ultimately received a mechanical AVR. The mean age of this patient cohort was 11 years and therefore comparable to our study cohort.

For our analysis, we used a conservative estimation of thromboembolic complications in mechanical AVR of 1.5% per year [6]. However, in a 30-year single-centre retrospective analysis at the University of Vienna, Schlein *et al.* found a considerably higher risk for thromboembolic and bleeding events with a composite linearized event rate per valve-year of 3.2% [22]. Had our study been based on this event rate, mechanical AVR would not have shown lower adverse events than Ross procedures.

To our understanding, whether a mechanical AVR is the better option remains unclear based on the data currently available. The decision requires individual assessment for each patient considering anatomical and myocardial factors, but also individual and familial risk factors for thromboembolic or bleeding events. Female adolescent patients are a subgroup, who may benefit from DAH implantation for later family planning reasons.

Subcoronary implantation of DAH could provide a future avenue to avoid full-root replacement and coronary artery reimplantation in patients with sufficient valvular dimensions. We have already begun pre-clinical work using respective animal models to explore this option.

In conclusion, current results for paediatric DAH are not ideal and do not fulfil the expectations of complete integration. They seem to elicit a low-grade immunologic reaction leading to valve degeneration, which requires reoperation.

This large prospective analysis, however, also demonstrates excellent mid-term survival with DAH and adverse event rates, which for the still limited data available give no reason to suspect inferiority to the results of paediatric Ross procedures.

### Limitations

The limited follow-up available for paediatric DAH patients and the overall number of patients treated so far is clearly an important limitation, and we want to stress that definitive conclusions will require a longer follow-up period.

This analysis compares prospectively acquired multicentre data for DAH with large meta-analyses based predominantly on retrospectively conducted single-centre reports and may be potentially influenced by currently unknown confounders. The strengths of the study include the relatively large size of the study group, the unique size of the comparative groups for AVR and the good age-matching between the groups. A limitation is presented by the lower degree of previous cardiac operations in the comparison groups.

## Data Availability

The data underlying this article will be shared on reasonable request to the corresponding author.

## Notes

Presented at the 2023 EACTS in Vienna, Austria. Registered under ClinicalTrials.gov, NCT02527629 (“Aortic Replacement Using Individualised Regenerative Allografts—ARISE the Surveillance”).

## ABBREVIATIONS

AVR: Aortic valve replacement
DAH: Decellularized aortic homografts
IQR: Interquartile range
LCx: Left circumflex artery

## References

[1] Saleeb SF , Gauvreau K, Mayer JE, Newburger JW. Aortic valve replacement with bovine pericardial tissue valve in children and young adults. Circulation 2019;139:983–5. 30742533 10.1161/CIRCULATIONAHA.118.037187

[2] Martin E , Laurin C, Jacques F, Houde C, Cote JM, Chetaille P et al More than 25 years of experience with the Ross procedure in children: a single-center experience. Ann Thorac Surg 2020;110:638–44. 31881194 10.1016/j.athoracsur.2019.10.093

[3] Zimmermann C , Attenhofer Jost C, Pretre R, Mueller C, Greutmann M, Seifert B et al Mid-term outcome of 100 consecutive Ross procedures: excellent survival, but yet to be a cure. Pediatr Cardiol 2018;39:595–603. 29327146 10.1007/s00246-017-1798-z

[4] Donald JS , Wallace FRO, Naimo PS, Fricke TA, Brink J, Brizard CP et al Ross operation in children: 23-year experience from a single institution. Ann Thorac Surg 2020;109:1251–9. 31863757 10.1016/j.athoracsur.2019.10.070

[5] Rowe G , Gill G, Zubair MM, Roach A, Egorova N, Emerson D et al Ross procedure in children: the Society of Thoracic Surgeons Congenital Heart Surgery Database Analysis. Ann Thorac Surg 2023;115:119–25. 35870519 10.1016/j.athoracsur.2022.06.043

[6] Etnel JR , Elmont LC, Ertekin E, Mokhles MM, Heuvelman HJ, Roos-Hesselink JW et al Outcome after aortic valve replacement in children: a systematic review and meta-analysis. J Thorac Cardiovasc Surg 2016;151:143–52.e1–3. 26541831 10.1016/j.jtcvs.2015.09.083

[7] Varrica A , Giamberti A, Lo Rito M, Reali M, Hafdhullah M, Satriano A et al Ross operation in pediatric population: impact of the surgical timing and the native pulmonary diameter on the outcome. Pediatr Cardiol 2023;44:663–73. 35994068 10.1007/s00246-022-02990-1

[8] Kallio M , Pihkala J, Sairanen H, Mattila I. Long-term results of the Ross procedure in a population-based follow-up. Eur J Cardiothorac Surg 2015;47:e164–70. 25661074 10.1093/ejcts/ezv004

[9] Brown JW , Patel PM, Ivy Lin JH, Habib AS, Rodefeld MD, Turrentine MW. Ross versus non-Ross aortic valve replacement in children: a 22-year single institution comparison of outcomes. Ann Thorac Surg 2016;101:1804–10. 27041455 10.1016/j.athoracsur.2015.12.076

[10] Etnel JRG , Grashuis P, Huygens SA, Pekbay B, Papageorgiou G, Helbing WA et al The Ross procedure: a systematic review, meta-analysis, and microsimulation. Circ Cardiovasc Qual Outcomes 2018;11:e004748. 30562065 10.1161/CIRCOUTCOMES.118.004748

[11] Binsalamah ZM , Ibarra C, Spigel Z, Zea-Vera R, Zink J, Heinle JS et al Primary aortic root replacement outcomes and risk factors in pediatric patients. Ann Thorac Surg 2020;110:189–97. 32251661 10.1016/j.athoracsur.2020.02.060

[12] Coti I , Wenda S, Andreeva A, Kocher A, Laufer G, Fischer G et al Donor-specific HLA antibodies after fresh decellularized vs cryopreserved native allograft implantation. HLA 2020;96:580–8. 32975376 10.1111/tan.14077 PMC7702054

[13] Ebken JMN , Smart I, Ramm R, Goecke T, Jashari R, Böthig D et al Residual immune response towards decellularized homografts may be highly individual. Eur J Cardiothorac Surg 2021;59:773–82. 33544830 10.1093/ejcts/ezaa393 PMC8083949

[14] Cvitkovic T , Bobylev D, Horke A, Avsar M, Beerbaum P, Martens A et al 4D-flow cardiac magnetic resonance imaging after aortic root replacement with long-valved decellularized aortic homografts: comparison to valve-sparing aortic root replacement and healthy controls. Eur J Cardiothorac Surg 2022;61:1307–15. 35079774 10.1093/ejcts/ezac016 PMC9154340

[15] Horke A , Bobylev D, Avsar M, Meyns B, Rega F, Hazekamp M et al Paediatric aortic valve replacement using decellularized allografts. Eur J Cardiothorac Surg 2020;58:817–24. 32443152 10.1093/ejcts/ezaa119 PMC7890932

[16] Cebotari S , Tudorache I, Ciubotaru A, Boethig D, Sarikouch S, Goerler A et al Use of fresh decellularized allografts for pulmonary valve replacement may reduce the reoperation rate in children and young adults: early report. Circulation 2011;124:S115–23. 21911800 10.1161/CIRCULATIONAHA.110.012161

[17] Notenboom ML , Schuermans A, Etnel JRG, Veen KM, van de Woestijne PC, Rega FR et al Paediatric aortic valve replacement: a meta-analysis and microsimulation study. Eur Heart J 2023;44:3231–46. 37366156 10.1093/eurheartj/ehad370 PMC10482570

[18] Sarikouch S , Theodoridis K, Hilfiker A, Boethig D, Laufer G, Andreas M et al Early insight into in vivo recellularization of cell-free allogenic heart valves. Ann Thorac Surg 2019;108:581–9. 30928547 10.1016/j.athoracsur.2019.02.058

[19] Oripov F , Ramm R, Falk C, Goecke T, Ebken J, Jashari R et al Serial assessment of early antibody binding to decellularized valved allografts. Front Cardiovasc Med 2022;9:895943. 36017105 10.3389/fcvm.2022.895943 PMC9395941

[20] Chakraborty J , Roy S, Ghosh S. Regulation of decellularized matrix mediated immune response. Biomater Sci 2020;8:1194–215. 31930231 10.1039/c9bm01780a

[21] Myers PO , Mokashi SA, Horgan E, Borisuk M, Mayer JE Jr, Del Nido PJ et al Outcomes after mechanical aortic valve replacement in children and young adults with congenital heart disease. J Thorac Cardiovasc Surg 2019;157:329–40. 30557950 10.1016/j.jtcvs.2018.08.077

[22] Schlein J , Simon P, Wollenek G, Base E, Laufer G, Zimpfer D. Aortic valve replacement in pediatric patients: 30 years single center experience. J Cardiothorac Surg 2021;16:259. 34496905 10.1186/s13019-021-01636-2 PMC8425048

